# The changes and its significance of Th17 and Treg cells and related cytokines in patients with tuberculosis pleurisy

**DOI:** 10.1186/1710-1492-10-28

**Published:** 2014-06-05

**Authors:** Guo-qiang Wang, Cai-ling Yang, Dong-fang Yue, Li-hong Pei, Hua Zhong, Ju-xia Niu

**Affiliations:** 1 The Clinical Laboratory of the First Affiliated Hospital of XinXiang, Medical University, Weihui City 453100, Henan, China; 2 Department of Oral and Maxillofacial Surgery, the First Affiliated Hospital of Xinxiang Medical University, Weihui City 453100, Henan, China; 3 The Clinical Laboratory of the Second People’s Hospital of Xinxiang, Xinxiang City 453000, Henan, China; 4 The Central Pharmacy of Infectious Disease Hospital of Xinxiang, Xinxiang City 453000, Henan, China

**Keywords:** Th17 cell, Treg cell, Tuberculous pleurisy, Flow cytometry, Cytokine

## Abstract

**Background:**

Tuberculous pleurisy is a kind of tuberculosis, it is well known that Th1 lymphocytes play a key role in the treatment of tuberculosis infection. However, latest studies show that Th17 lymphocyte may also play an important role tuberculosis infection. There is close relationship between Treg and Thl7 cells, and changes in the number or the function of the two kinds of cells may lead to diseases. The current researches on Thl7 and Treg cells maily focus on autoimmune diseases, however, reports about their role in tuberculosis are limited. In this study, we investigate the function of th17 and Treg cells and the above cytokines in the pathogenesis of tuberculosis pleurisy; by determining the expression of Th17 and Treg cells in peripheral CD4 T cells and the related cytokines in patients with tuberculous compared with healthy people.

**Results:**

Th17 cells in patients were higher than that in the Healthy control group, expression of Treg cells in patients were lower than that in the healthy group; IL-17, IL-23 levels in peripheral blood and hydrothorax from the patients were higher than that in the healthy group; IL-17, IL-23 and IL-6 levels in hydrothorax were higher than that in peripheral blood. There was no difference in IL-6 level in peripheral blood between the patients and healthy control; TGF- β level in peripheral blood from the healthy group was higher than that in peripheral blood and hydrothorax from the patients. And there were no differences in TGF- β level between peripheral blood and hydrothorax. Th17 cells were negatively correlated with Treg cells ,but were positive correlation with IL-17, IL-23, IL-6 levels in peripheral blood; TGF- β level was positive correlation with Treg cells in the peripheral blood, but no correlation with Th17 cells.

**Conclusion:**

Th17 and Treg cells may be involved in the immune pathological mechanism of tuberculous pleurisy and changes of related cytokines may be involved in the differentiation of Th17 and Treg cells and inflammatory response. Thus, Th17 and Treg cells and related cytokines may be important immunopathogenesis for tuberculous pleurisy.

## Background

Tuberculosis is one of the most important infectious diseases worldwide [1]. In China, tuberculosis also poses a very serious threat to health [2]. Tuberculous pleurisy is a kind of tuberculosis. It is believed that Th1 lymphocytes play a key role in the treatment of tuberculosis infection [3,4]. Aside from Th1 lymphocytes, latest studies have shown that Th17 lymphocyte may also play a very important role in fighting against tuberculosis infection [5,6]. Th17 cells are a type of immune cells discovered in recent years. They are CD4 + T lymphocyte subsets, which are different from the Thl and Th2 immunoreactive cells. Thl7 and Treg cells are closely related with each other. Though they are functionally antagonistic, they are correlated during the process of differentiation.

Changes in the number or in the function of the two kinds of cells may lead to diseases. The current research on Thl7 and Treg cells was more or less concentrated on autoimmune diseases. Rarely is seen the reports about their role in tuberculosis. Among the limited number of studies on the subject, the views on the role of Thl7 cells and Treg cells in tuberculosis do not agree with each other, which we will discuss in detail in the discussion part. For the above reasons, we detected Th17 and Treg cells and the related cytokines in patients with tuberculous pleurisy and those in the healthy to investigate the roles of Th17 and Treg cells and the related cytokines in immunopathogenesis of tuberculous pleurisy.

## Materials and methods

### Subjects

All subjects are outpatients without drug treatment in the Tuberculosis Department of the First Affiliated Hospital of XinXiang Medical University from January 2011 to December 2012 (this experimental research is approval of the ethics committee of Xinxiang medical university), 34 healthy cases were used as the control group, with mean age 33.6 ± 8.7 years old. Among them, 24 were males and 10 were females. 35 tuberculous pleurisy cases were used as the experimental group, mean age 30 ± 7.8 years old. 26 of them were males and 9 were females. The above tuberculosis cases were in line with China’s TB diagnosis standard, while physical examination showed that there were no other pathogen infections and autoimmune diseases in them.

### Experimental method

6 ml of blood was collected from the veins of each case in both the experimental and the control groups. Blood was divided into two tubes, 3 ml in each tube. One tube is for the detection of Thl7 and Treg cells by flow cytometry. The other tube was for quantitative detection of IL-17, IL-23, IL-6, TGF- β factor. 5 ml of hydrothorax were extracted from all the patients for quantitative detection of IL-17, IL-23, IL-6, TGF- β factor by ELSIA method. The operation was in strict accordance with the instructions.

### Instruments and reagents

Instruments: the flow cytometry was from BD company (FACS Canto) in USA. The enzyme immunoassay instrument was from the Aidekang Company in Yantai, Shandong Province, China; Reagents: Thl7 and Treg kits were from Biolegend company. Thl7 kits include FITC labeled anti human CD3, PE labeled anti human CD4, Alexa 647 Fluor markers anti human IL-7 and the matched rat IgGl isotype control, stationary liquid and membrane rupture lotion. And the Treg kits include FITC labeled anti human CD4, PE labeled anti human CD25, Alexa Fluor647 FOXP3 and the matched rat IgGl isotype control, Foxp + 3 fixed/rupture of membrane liquid and rupture of membrane buffer. Phorbol ester and anionomycin were purchased from Sigma company. Mo can mycin was purchased from Biolegend company. IL-17 kit was from Biosource company. IL-23 kit was from Bender Medsestems Company. IL-6 and TGF- β kit were from Xinbosheng Biological Technology Co. in China.

### Statistical analyses

Data were analyzed by SPSS17.0 statistical software. All data were indicated as means and standard deviations (x ± s). The statistic difference among means of experimental data were measured by independent sample T test and paired data T test, and correlation analysis was carried out by Pearson correlation analysis. And P < 0.05 is regarded as statistically significant.

Declaration: the involved patients in the research have agreed to publish manuscript.

## Results

### Expression of Th17 and Treg cells in CD4+ Tcells in peripheral blood

**Figure 1:**
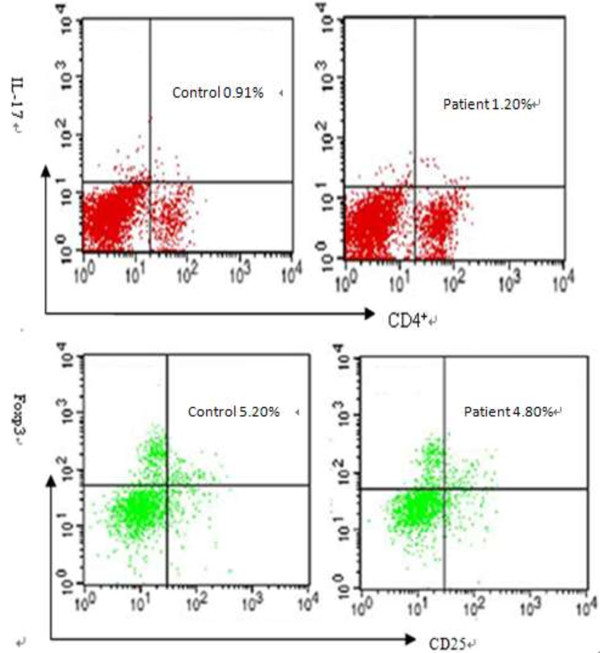
Flow cytometry pictures of Th17 and Treg cells.

**Figure 2:**
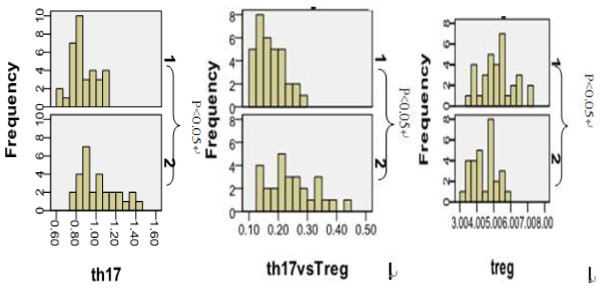
Distribution pictures of expression of Th17 and Treg cells (notes: 1. the control group 2. the patient group. The abscissa: expression ratios of cells; the ordinate: frequency of Corresponding ratios).

The percentage of Th17expression in CD4+ cells were 0.89 ± 0.13% in control group and 1.02 ± 0.20% in the experimental group. The expression of Th17 cells in patients group was significantly higher than in the control group, *P* < 0.05. Expression ratios of Treg cell were 5.10 ± 0.90% in control group and 4.64 ± 0.77% in patient group. The ratio of Treg cells in control group was significantly higher than that in patient group, *P* < 0.05. The ratio of Th17/Treg cells in patient group was significantly higher than in the control group, P < 0.05(Table 1). FCM pictures of Th17 and Treg cells in CD4+ cells in peripheral blood were shown in Figure 1, and distribution pictures of expression of Th17 and Treg cells in CD4+ cell in peripheral blood were shown in Figure 2.

**Table 1:** Expression of Th17 cells and Treg cells in peripheral blood in control and patient groups

**Groups**	**Cases**	**Th17cell (%)**	**Tregcell (%)**	**Th17cell/Tregcell**
Control	34	0.89 ± 0.13	5.10 ± 0. 90	0.17 ± 0.05
Tuberculous pleurisy	35	1.02 ± 0.20	4.64 ± 0.77	0.25 ± 0.07

### Change of cytokines level in patients with tuberculous pleurisy

**Figure 3:**
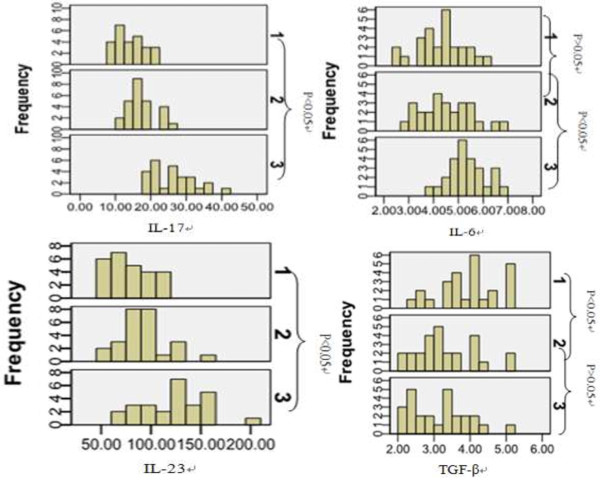
Frequency of various cytokines level in peripheral blood and hydrothorax (notes: 1. cytokines level in blood from the healthy; 2. cytokines level in blood from the patients; 3. cytokines level in hydrothorax from the patients. The abscissa: cytokines level; the ordinate: frequency of corresponding cytokines).

IL-17 and IL-23 levels in peripheral blood from patients with tuberculous pleurisy were significantlyhigher than those from the healthy, the *P* values being 0.022 and 0.039 (the independent sample T test). IL-17 and IL-23 levels in hydrothorax from were significantly higher than that in the blood from the patients (paired data T test), with the *P =*0.000 and 0.000; IL-6 level in hydrothorax from the patients was significantly higher than that in the blood from patients (T test for paired data) and in the blood from the healthy (independent samples T test), *P =*0.003 and 0.000. There was no difference in IL-6 level between blood from patient and that from the healthy (independent samples T test, *p* = 0.274). TGF-βconcentration in peripheral blood from the healthy was significantly higher than in peripheral blood and hydrothorax from the patients (independent samples T test), with =0.005 and 0.000 respectively. There was no significant difference between TGF- β level in blood and in hydrothorax from the patients, p = 0.365 (paired data T test) (Table 2). The cytokine level distribution was shown in Figure 3 (Notes: 1: The cytokines in peripheral blood from the healthy; 2: the cytokines in peripheral blood from the patients; 3: cytokines in hydrothorax from the patients).

**Table 2:** Cytokines level in periphery blood from the healthy and the patients and hydrothorax from the patients

**Group**	**Cases**	**IL-17**		**IL-6**	
		**Peripheral blood**	**Hydrothorax**	**Peripheral blood**	**Hydrothorax**
Control	34	14.45 ± 3.81		4.26 ± 0.91	
Tuberculous pleurisy	35	17.49 ± 3.94	26.13 ± 5.98	4.54 ± 1.02	5.31 ± 0.74
**Group**	**Cases**	**IL-23**		**TGF-β**	
		**Peripheral blood**	**Hydrothorax**	**Peripheral blood**	**Hydrothorax**
Control	34	77.55 ± 20.26		3.95 ± 0.79	
Tuberculous pleurisy	35	90.42 ± 23.06	122.26 ± 31.71	3.32 ± 0.80	3.12 ± 0.77

### The correlation among Th17 and Treg cells and cytokines in peripheral blood

Th17 cells and IL-17, IL-23, IL-6 were positively correlated, *P* < 0.05. But there was no significant correlation between Th17 cells and TGF-β level, P > 0.05. Treg cells and IL-17, IL-23 were of significant negative correlation. There was a significant positive correlation between Treg cells and TGF- β, P < 0.05 (Table 3).

**Table 3:** The correlation among Th17 and Treg cells and cytokines in peripheral blood

**Immune cell**	**Th17cell**		**Tregcell**	
**Cytokines**	**r**	**p**	**r**	**p**
IL-17	0.479	0.013	−0.356	0.016
IL-23	0.441	0.015	−0.318	0.019
IL-6	0.326	0.017	0.152	0.089
TGF-β	0.091	0.659	0.297	0.024

## Discussion

Th17 cells are a type of immune cells found in recent years. They are CD4 + T cell subsets with strong proinflammatory effect,but they differ from Thl and Th2 immune cells [7]. Thl7 cells and Treg cells are closely related to each other. Though functionally antagonistic, they are correlated in the process of differentiation. Changes in their number or in their ways of functioning can lead to the disease. The current research on Thl7 cells and Treg cells is more concentrated on their roles in autoimmune diseases, but their roles in tuberculosis germination and development remain unclear [8,9]. Recently, it is believed that Th1 cells and IFN- γ play a very important role in the protective immunoreaction against tuberculosis [3,4]. But besides Th1 cells, other immune cells and cytokines could also be important for fighting against tuberculosis [5,6,8]. So we designed the study to explore roles of Thl7 and Treg cells and the related cytokines in pathogenesis of tuberculosis. In this study, Thl7 cells from patients with tuberculous pleurisy were significantly higher than from the healthy, but Treg cells were lower than from the healthy, and the ratios of Thl7/Treg from patients were significantly higher than from the healthy, which showed immune imbalance between Th17 cells and Treg cells from the patients. The imbalance may play an important role in the pathogenesis of tuberculosis.At present, the roles of Thl7 and Treg cells in tuberculosis were rarelyreported. Even when reported, views varied greatly from researcher to researcher. Previous reports showed that Thl7 cells from patient with tuberculosis were higher than those from the healthy [5,6,10], and that Treg cells were significantly lower than those from the healthy [10], which was in line with our results. But others reported contradictory research results [1,11]. Some showed that the expression ratio of Thl7 cells in tuberculosis patients didn’t vary significantly from those in the healthy [12], or it was even lower than in the healthy when accompanied by less expression of IL-6R of CD4 + T cells [13]. Th17 cells were considered to be one of the most important cells to mediate inflammation and autoimmunity action [14], and played important roles in autoimmune diseases, in fighting against extracellular bacteria infection, in mediating chronic inflammation and tumor. Thl7 cells mainly secreted IL-17A, IL-17 F, IL-6 and tumor necrosis factor (TumorNecrosis, factor, TNF) - α, IL-21, IL-22, and macrophage colony stimulating factor (granulocyte –macrophage colony -stimulating factor, GM-CSF). Treg cells were a kind of CD4 + T cells with specific expression of transcription factor Foxp3 and negative immune regulatory function. They could exert anti-inflammatory effects or maintain auto-immune tolerance by contact inhibition of cells and by secreting inhibitory cytokines, such as lL-10 and TGF- β. Thl7 cells were closely related with Treg cells. Both were differentiated from natural CD4 + T cells, and were related and diversionary in their differentiation and function [15]. In the present study, Th17 cells from untreated patients with tuberculosis pleurisy were significantly increased, while Treg cells decreased, which showed that the unbalance between Th17 and Treg cells played an important role in immunopathogenesis of tuberculous pleurisy. But why there was inconsistency between our research and previous reports? We think the following factors may be important: 1. The varied immunity of the selected experimental subjects may play a part in the inconsistency; 2. Different sampling time may also be an important factor. Sampling from subjects may happen before treatment, after treatment or during treatment, which could also lead to the discrepancy in results; 3. Choice of different experimental methods also had certain influence on the results. IL-23 is an important cytokine that takes part in differentiation of Th17 cells [16,17]. It is not the initiation factor of Th17 cell differentiation, but it is an important survival factor of Th17 cell. IL-23 was produced by macrophages enabled by antigen in the process of inflammation [18]. In the present study, IL-23 level in peripheral blood and hydrothorax from the patients was significantly higher than in peripheral blood from the healthy, and there was a positive correlation between IL-23 level and Th17 cells level in peripheral blood, which shows that IL-23 plays a role in the increase of Th17 cells.

Th17 cells produced a cytokine, IL-17. By combining with IL-17 receptors on epithelial cells, endothelial cells, and fibroblasts, IL-17 could activate MAP kinase and NF-kB so that they could exert their biological activity [15], which enabled these cells to produce a series of cytokines to guide leukocytes to gather at the infectious part where these leukocytes became mature and activated, and released lysosomal enzymes that could cause injury to endothelial cells and relating tissues, thus aggravating inflammation. IL-17 could also lead to macrophage accumulation in inflamed areas. These macrophages were activated by bacteria and the toxins produced by bacteria. The activated macrophages were involved in inflammation and immune response, and produced IL-6, IL-1, TNF-a, and IL-23, which enhanced local inflammation. The produced IL-23 promoted the differentiation of Th17 cells and helped maintain Th17 cells at a high level. The elevated Th17 cells produced more IL-17 which aggravated inflammation reaction. In this study, Th17 cells were significantly positively correlated to IL-17 level in peripheral blood and hydrothorax from the patients, which confirmed that IL-17 was produced by Th17 cells. Although IL-23 and IL-17 showed a positive correlation with Th17 cells, they were significantly negatively correlated with Treg cells. The negative relation might indicate that IL-23 and IL-17 inhibited Treg cells, and some previous researches indicated that IL-23 and IL-17 could inhibit the function of Treg cells [19,20]. But we found no theoretical basis to support that idea. IL-6 is a cytokine secreted by Th17 cells. In this study, IL-6 levels in peripheral blood and hydrothorax from the patients were significantly higher than in peripheral blood from the healthy. There was a significant positive correlation between IL-6 levels and Th17 cells, but no significant correlation between IL-6 levels and Treg cells. TGF-β is a cytokine secreted by Treg cells. In this study, there was no significant correlation between TGF-β and TH17cells in peripheral blood, and there was a significant positive correlation between TGF-β and Treg cells in peripheral blood. TGF-β in peripheral blood from the healthy was higher than in peripheral blood and hydrothorax from the patients, which showed that TGF-β was from Treg cells. Research showed that IL-6 was a key factor connecting Th17 and Treg cells [21]. When TGF- β alone was stimulated activated CD4+ cells, the activated CD4 + cells differentiated into Treg cells. But when TGF-β and IL-6 were stimulated together, the activated CD4 + cells would differentiate into TH17 cells [22]. Based on the above evidence, it could be concluded that Th17 cells would further increase because of the high level of IL-6 with a certain concentration of TGF- β in inflammation part, therefore increasing the inflammation reaction of patients.

In summary, the unbalance between Th17 and Treg cells played an important role in the immune pathological injury of patients with tuberculous pleurisy. Excess IL-17, IL-6, and IL-23 in inflammation part further increased the number of Th17 cells. Increased Th17 cells secreted large amounts of inflammatory cytokines. Then these inflammatory cytokines increased inflammatory reaction of inflammatory part. So the unbalance between TH17 and Treg cells and the inflammatory cytokines secreted by TH17 and Treg cells play important roles in the immune pathological reaction of patient with tuberculous pleurisy. The research results may provide a theory support for the treatment of tuberculous pleurisy.

## Abbreviations

Th17: T help cell 17; Th1: T help cell 1; Th2: T help cell 2; Treg cell: Regulatory T cells; IL-17: Interlukine 17; IL-23: Interlukine 23; IL-6: Interlukine 6; TGF-β: Transforming growth factor-β.

## Competing interests

The authors declare that they have no competing interests.

## Authors’ contributions

GQ W conceived and designed the study and wrote the manuscript. LHP and HZ and DF Y performed all laboratory work, compiled the data. CL Y and JX N performed the statistical analysis and initial interpretation of results and helped to draft the manuscript. All authors read and approved the final manuscript.
